# Dynamic Antibody Responses in Porcine Epidemic Diarrhea Virus‐Infected Pigs and Correlation of Prepartum Serum, Oral Swabs, and Rectal Swabs With Postpartum Colostral IgA and IgG in Sows

**DOI:** 10.1155/tbed/6411953

**Published:** 2026-05-26

**Authors:** Ping Chen, Wenlong Zhao, Ruide Deng, Jie Fan, Mei Huang, Yirun Tai, Mengyao Lu, Zhongxin Fan, Runcheng Li, Wei Dong, Xiaoming Yuan, Meng Ge

**Affiliations:** ^1^ College of Veterinary Medicine, Hunan Agricultural University, Changsha, 410125, China, hunau.edu.cn; ^2^ Zhangjiajie College, Zhangjiajie, 427000, China; ^3^ Hunan Animal Disease Prevention and Control Center, Changsha, 410128, China

## Abstract

Porcine epidemic diarrhea (PED) is a highly contagious gastrointestinal disease caused by PED virus (PEDV), resulting in high mortality among neonatal piglets. Ensuring robust maternal immunity to provide protective colostral antibodies is critical for effective disease control. In this study, an enzyme‐linked immunosorbent assay (ELISA) was employed to monitor antibody dynamics in PEDV‐infected pigs, revealing long‐term serum antibody persistence. PEDV‐specific immunoglobulin (Ig)A and IgG levels were subsequently measured in prepartum serum, oral and rectal swabs, and postpartum colostrum from sows administered with different immunization strategies, including commercial vaccination and feedback. Correlations between the antibody levels in these sample types and colostrum were analyzed in order to facilitate estimation of maternal immunity and prompt adjustment of immunization programs. Results revealed that PEDV‐specific IgA and IgG in piglets seroconverted 1 week after infection, peaked at two to 3 weeks, and remained detectable up to 31 weeks. Analysis of 1159 samples collected from sows at various stages across four commercial farms revealed strong correlations between serum and colostrum PEDV IgA and IgG. After the first immunization, the highest correlation coefficients for IgA and IgG were 0.6898 (*p* < 0.0001) and 0.5959 (*p* < 0.0001), respectively. After the second immunization, peak correlations reached 0.7239 (*p* < 0.0001) for IgA and 0.6051 (*p* < 0.0001) for IgG. On the day of farrowing, correlations were strongest, with IgA reaching 0.8178 (*p* < 0.0001) and IgG reaching 0.8682 (*p* < 0.0001). Additionally, oral and rectal swab IgA exhibited correlations with colostral IgA, reaching peak *r* values of 0.6542 (*p* < 0.0001) and 0.6347 (*p* < 0.0001), respectively, but showed minimal correlation with colostral IgG. These findings indicate that, in clinical practice, measuring PEDV‐specific IgA in sow serum can provide a reliable assessment of maternal immune status and vaccine efficacy, while oral swabs and rectal swabs offer an auxiliary diagnostic avenue for supplementary evaluation. This approach facilitates earlier, more precise adjustments to immunization programs and provides a theoretical basis for optimized PEDV prevention and control strategies.

## 1. Introduction

Porcine epidemic diarrhea (PED) is an acute and highly contagious enteric disease caused by PED virus (PEDV). This disease is characterized by high morbidity and rapid transmission, and piglets of all ages are susceptible, although clinical manifestations are most severe in neonates [1]. PEDV was first identified in Europe in the early 1970s and subsequently spread to North America and Asia. Ongoing viral mutations and dissemination have resulted in severe economic losses to the swine industry [1–4]. PEDV is an enveloped, single‐stranded, positive‐sense RNA virus of the genus Coronavirus, encoding four structural proteins: spike (S), envelope (E), membrane (M), and nucleocapsid (N) [5]. Among these, the S protein, located on the viral surface, is the primary target of neutralizing antibodies and plays essential roles in receptor binding and membrane fusion [6, 7]. Following infection, PEDV replicates within villus epithelial cells, damaging cellular structures and causing villous atrophy, impaired nutrient absorption, diarrhea, cellular degeneration, necrosis, and viral shedding [8, 9]. In sows, PEDV infection stimulates mucosal intestinal immunity, inducing immunoglobulin (Ig)G and IgA antibody production. These antibodies are subsequently transferred to the mammary gland via Fc receptors and the gut–mammary gland‐secretory IgA (sIgA) axis, respectively, and secreted into colostrum and milk [10], thereby providing piglets with essential Igs. The sIgA is the predominant antibody conferring PEDV protection. It adheres to the intestinal mucosa of piglets to provide mucosal immunity throughout lactation [11].

Existing studies have demonstrated that colostral IgA levels strongly correlate with PEDV protection, whereas high IgA levels can effectively prevent PEDV infection in piglets [12]. However, high levels of IgG can only alleviate certain clinical symptoms and improve piglet survival rates but cannot provide complete protection against PEDV [13]. Commercial inactivated or attenuated vaccines can induce high IgG levels in sows but fail to elicit sufficient IgA production [14], leaving piglets partially unprotected. When commercial vaccines are inadequate, some farms use alternative strategies, such as inactivated tissue vaccines derived from diseased pigs or feedback to control PED. Feedback is a measure used to control PED, which involves feeding the gastrointestinal tract materials or diarrheic feces from infected pigs to uninfected sows or replacement gilts [15]. Current clinical evaluation of piglet immune protection against PEDV primarily relies on colostral antibody level measurement. However, this retrospective approach delays immune assessment, leaving sows with insufficient antibody levels without timely booster immunization or intervention, which can lead to piglet morbidity and mortality. Therefore, developing a reliable method to assess PEDV vaccine‐induced immunity in sows before farrowing is critical.

Previous reports indicate a moderate correlation (*r* ≈ 0.6) between PEDV‐specific IgA antibodies in serum collected from sows 7 days before farrowing and on the farrowing day and those in colostrum, indicating that serum antibody levels can partially reflect colostral antibody levels [16, 17]. However, these studies did not compare the correlation between serum and colostrum samples across different stages of the immunization program. Ouyang et al. [18] reported that PEDV‐specific IgA antibodies could be detected in the oral fluids and feces of primiparous and sows, but their correlation with PEDV antibodies in colostrum remains unclear. Therefore, this study systematically analyzed PEDV‐specific IgA and IgG correlations among serum, oral swabs, rectal swabs, and colostrum from sows under diverse immunization backgrounds to identify a timely and effective optimal method for immune evaluation. Crucially, the utility of any prepartum predictive tool depends on the precondition that vaccine‐ or infection‐induced antibodies persist at detectable levels throughout the prepartum period. To first establish this premise, we investigated the long‐term antibody kinetics in PEDV‐infected piglets. Subsequently, building upon this foundation, we conducted a systematic correlation analysis between prepartum samples and postpartum colostrum in sows. This comprehensive approach aimed to provide a basis for earlier immune assessment and to address a gap in the understanding of long‐term PEDV immunology.

## 2. Materials and Methods

### 2.1. Virus Strain and Preparation

The PEDV strain used was CH‐HNZZ‐2023 (GenBank: PV464256), isolated from clinical cases by our laboratory and stored at −80°C. Its 50% tissue culture infectious dose (TCID_50_) was 10^3.74^ TCID_50_/100 μL. For experimental use, commercially available sterile milk was utilized as a diluent to prepare viral suspensions at 10^4^ TCID_50_/mL.

### 2.2. Piglet Infection and Sample Collection

Twelve 30‐day‐old weaned piglets, confirmed negative for PEDV antigen and antibodies, were selected for the study and randomly assigned to a PEDV‐infected group (*n* = 6) and a negative control group (*n* = 6). Piglets in the PEDV‐infected group were orally inoculated with PEDV at 10^4^ TCID_50_/kg, whereas those in the control group were orally administered an equivalent volume of commercially available sterile milk to serve as a mock‐infected control using the identical handling procedure. The animals were observed over 31 weeks with serum antibody levels monitored continuously.

Blood samples were collected from the anterior vena cava once weekly for the first 7 weeks postinfection and biweekly thereafter. The serum was separated by centrifugation and analyzed to determine PEDV antibodies using an enzyme‐linked immunosorbent assay (ELISA). The infection and control groups were housed in isolation at 25 ± 2°C and 70% ± 5% relative humidity.

### 2.3. Sample Collection for Antibody Correlation Analysis in Sows

Serum and colostrum samples were collected from four large‐scale pig farms (designated A, B, C, and D) in Hunan Province, China, each of which employed distinct PEDV immunization programs. Additionally, oral swabs and rectal swabs were collected from sows in Farms A and B. The immunization protocol of Farm A comprised two doses of a live‐attenuated vaccine on days 35 and 21 before farrowing. Piglets from this farm remained PEDV‐free. Farm B implemented feedback 180 days before farrowing, followed by two doses of an inactivated tissue vaccine at 28 and 14 days prefarrowing. Piglets were infected during the nursery phase, with a mortality rate of less than 4%. Farm C combined an immunization strategy that involved a live‐attenuated vaccine at 50 days before farrowing, an inactivated vaccine at 32 days before farrowing, and an inactivated vaccine plus feedback at 19 days before farrowing; piglets in this farm were infected with PEDV before weaning at ~15–21 days of age, with a mortality rate of less than 10%. Farm D did not administer vaccination, and sows were naturally infected with PEDV during late gestation. Piglets exhibited infection and diarrheal symptoms within 7 days of birth, with a mortality rate of ~50%. Table 1 presents the detailed information on all collected samples.

**Table 1:** Collection time, types, and quantities of samples from various farms.

Farm	Collection time	Type and quantity			
		Serum	Colostrum	Oral swabs	Rectal swabs
Farm A	First immunization day	40	/	40	40
	Second immunization day	40	/	40	40
	Farrowing day	40	40	40	40
Farm B	First immunization day	20	/	57	57
	Second immunization day	20	/	57	57
	Farrowing day	44	80	68	81
Farm C	First immunization day	50	/	/	/
	Farrowing day	50	50	/	/
Farm D	Farrowing day	34	34	/	/

*Note:* “/” denotes no sample collected for the corresponding type.

### 2.4. Serum Sample Processing and Storage

We collected 3–4 mL of blood from the anterior vena cava using vacuum non‐anticoagulant blood collection tubes (KWS, China). The samples were kept at room temperature for 4 h to allow serum separation and subsequently centrifuged at 3500 × *g* for 5 min. The resulting serum was aliquoted and stored at −20°C until further use.

### 2.5. Oral Swabs Processing and Storage

Sterile swabs were inserted into the oral cavity of pigs and rotated several times to collect saliva. Each swab was subsequently placed into a centrifuge tube containing 1 mL of sample diluent with a stabilizing agent and vortexed vigorously for 5 min. The swabs were left undisturbed at 4°C for 3 h and subsequently centrifuged at 10,000 × *g* for 5 min. The resulting supernatant was collected and stored at −20°C until further use.

### 2.6. Rectal Swabs Processing and Storage

Sterile swabs were inserted 5–10 cm into the rectum of pigs and rotated several times to collect fecal material. Swabs were subsequently placed into centrifuge tubes containing 1 mL of sample diluent containing a stabilizing agent and vortexed vigorously for 5 min. The swabs were left undisturbed at 4°C for 3 h and subsequently centrifuged at 10,000 × *g* for 5 min. The resulting supernatant was collected and stored at −20°C until further use.

### 2.7. Colostrum Processing and Storage

The teats and udder of each sow were cleaned with distilled water, after which the udder was gently massaged and hand‐expressed to collect colostrum. The samples were transferred into 5 mL centrifuge tubes and stored at −20°C until further use.

### 2.8. ELISA Detection of PEDV IgA

PEDV‐specific IgA antibody levels were measured using an in‐house indirect ELISA (iELISA) method based on PEDV S1 protein [19]. Purified PEDV S1 protein was coated onto 96‐well ELISA plates (Costar, USA) at 50 ng per well. Serum samples were diluted 1:50, oral and rectal swabs at 1:1, and colostrum samples at 1:50. Because the OD_450_ nm values of serum and colostrum samples from Farm B exceeded the upper detection limit of the instrument, gradient dilution was performed. The optimal dilution ratios were determined to be 1:500 for serum and 1:10,000 for colostrum. After thorough mixing, 100 μL of each diluted sample was added to the antigen‐coated wells and incubated at 37°C for 30 min. The plates were washed thrice with phosphate‐buffered saline with Tween‐20 **(**PBST), followed by the addition of 100 μL of horseradish peroxidase (HRP)‐conjugated goat anti‐pig IgA antibody (SeraCare, USA) to each well and incubation at 37°C for 30 min. After three additional washes with PBST, 50 μL of tetramethylbenzidine (TMB; SeraCare, USA) substrate solution was added to each well and incubated at 37°C. The reaction was terminated with 50 μL of 2 M H_2_SO_4_, and absorbance was measured at OD_450_ nm using a microplate reader (Thermo Fisher Scientific, USA). The sample‐to‐positiveup][?tjl=1000][?down]? (S/P) ratio was calculated as follows:

SP=OD450sample−OD450negative sampleOD450positive sample−OD450negative sample.

### 2.9. ELISA Detection of PEDV IgG

PEDV‐specific IgG antibodies were detected following the same procedure described above, except that HRP‐conjugated goat anti‐pig IgG (SeraCare, USA) was used as the secondary antibody.

### 2.10. Statistical Analysis

Antibody levels were evaluated using the S/P ratio calculated from the OD_450_. Samples with an S/P ≥ 0.4 were considered positive, whereas those with an S/P < 0.4 were considered negative. Data normality was assessed using the Shapiro–Wilk test. As the antibody data did not follow a normal distribution, correlations among different sample types were analyzed using Spearman’s rank correlation. All statistical analyses were performed using GraphPad Prism 10.0. Correlation coefficients were reported as *r* values, with statistical significance set at *p* < 0.05. According to Chan [20], correlations were interpreted as *r* ≥ 0.8, very strong; 0.6 ≤ *r* < 0.8, moderately strong; 0.3 ≤ *r* < 0.6, fair; *r*＜0.3, poor.

## 3. Results

### 3.1. Dynamics of IgA and IgG Antibodies in PEDV‐Infected Piglets

**Figure 1:**
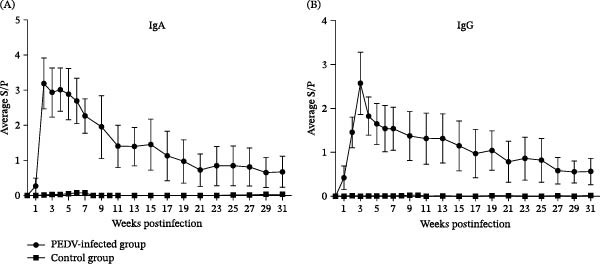
Dynamic changes of PEDV‐specific IgA (A) and IgG (B) in piglets following experimental infection (infection group, *n* = 6; control group, *n* = 6).

Piglets developed an anti‐PEDV antibody response 1 week after viral infection, as shown in Figure 1. Serum‐specific IgA and IgG levels peaked at weeks 2 and 3 postinfection, respectively, and subsequently declined, stabilizing around 20 weeks postinfection. Both antibodies remained detectable at week 31 postinfection. This observation indicates that PEDV infection can induce a long‐lasting antibody response.

### 3.2. PEDV‐Specific IgA and IgG Antibody Levels and Positivity Rates in Sows With Different Immunization Backgrounds

**Figure 2:**
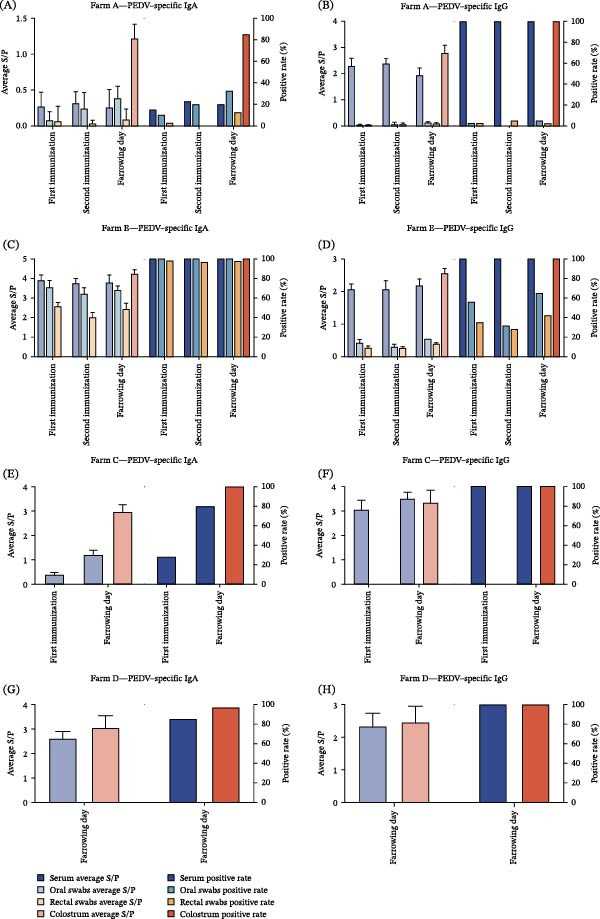
PEDV‐specific IgA and IgG antibody levels and positivity rates in different sample types under various immunization programs. (A, B) S/P values and positivity rates of IgA (A) and IgG (B) in different sample types from Farm A. (C, D) S/P values and positivity rates of IgA (C) and IgG (D) in different sample types from Farm B. (E, F) S/P values and positivity rates of IgA (E) and IgG (F) in different sample types from Farm C. (G, H) S/P values and positivity rates of IgA (G) and IgG (H) in different sample types from Farm D. Detailed sample sizes (*n*‐value) for each group are provided in Table 1.

PEDV‐specific IgA and IgG antibody levels and corresponding positivity rates in sows with different immunization backgrounds are presented in Figure 2. In Farm A (commercial live‐attenuated vaccine), PEDV‐specific IgA in serum and swabs remained consistently low (S/P range: 0.031–0.38) throughout the immunization periods and at farrowing day. In contrast, PEDV‐specific IgA in colostrum peaked at farrowing, with a positivity rate of 85%. PEDV‐specific serum IgG remained highly positive across all stages, while PEDV‐specific IgG in oral and rectal swabs was nearly undetectable throughout the study. In Farm B (feedback plus inactivated vaccine), high levels of PEDV‐specific IgA (S/P range: 2.01–4.24) and near‐100% positivity rates were maintained in all sample types from the first immunization to farrowing. PEDV‐specific serum IgG also remained strongly positive, whereas PEDV‐specific IgG in oral and rectal swabs showed moderate positivity (31.6%–64.9%). In Farm C (combined immunization strategy), a clear temporal upward trend was observed. The S/P values of PEDV‐specific serum IgA increased progressively from the first immunization to farrowing, with positivity rising from 28% to 80%. PEDV‐specific serum IgG remained consistently high at both stages. Both PEDV‐specific IgA and IgG in colostrum reached 100% positivity at farrowing. In Farm D (nonimmunized), the S/P values for both PEDV‐specific IgA and IgG exceeded 2.0 in serum and colostrum, with 100% positivity in both sample types.

### 3.3. Correlation Between Serum and Colostrum IgA Antibodies

**Figure 3:**
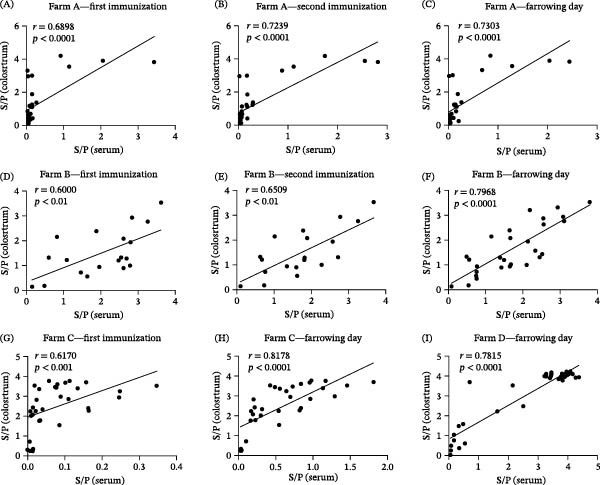
Correlation between serum and colostrum IgA antibodies in sows from different farms under various immunization regimens. Correlations of serum and colostrum IgA levels at the first immunization (A) (*n* = 40), second immunization (B) (*n* = 40), and the day of farrowing (C) (*n* = 40) in Farm A. Correlations of serum and colostrum IgA levels at the first immunization (D) (*n* = 20), second immunization (E) (*n* = 20), and the day of farrowing (F) (*n* = 44) in Farm B. Correlations of serum and colostrum IgA levels at the first immunization (G) (*n* = 50) and the day of farrowing (H) (*n* = 50) in Farm C. (I) Correlations of serum and colostrum IgA levels on the day of farrowing in Farm D (*n* = 34). “*r*” values represented correlation coefficients. “*p*” values meant statistical significance.

Serum and colostrum IgA levels exhibited a robust correlation across various immunization stages. At the first immunization, serum and colostrum IgA were moderately strongly correlated in Farm A (*r* = 0.6898, *p* < 0.0001), Farm B (*r* = 0.6000, *p* < 0.01), and Farm C (*r* = 0.6170, *p* < 0.001) (Figure 3A, D, G). During the second immunization, the correlation increased in Farm A (*r* = 0.7239, *p* < 0.0001) and Farm B (*r* = 0.6509, *p* < 0.01) (Figure 3B, E). On the day of farrowing, moderate to very strong correlations were observed in Farm A (*r* = 0.7303, *p* < 0.0001), Farm B (*r* = 0.7968, *p* < 0.0001), Farm C (*r* = 0.8178, *p* < 0.0001), and Farm D (*r* = 0.7815, *p* < 0.0001) (Figure 3C, F, H, I).

### 3.4. Correlation Between Serum and Colostrum IgG Antibodies

**Figure 4:**
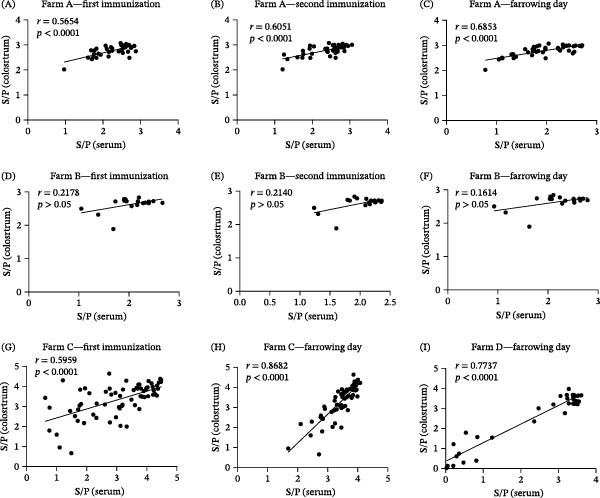
Correlation between serum and colostrum IgG antibodies at different immunization stages. Correlations of serum and colostrum IgG levels at the first immunization (A) (*n* = 40), second immunization (B) (*n* = 40), and the day of farrowing (C) (*n* = 40) in Farm A. Correlations of serum and colostrum IgG levels at the first immunization (D) (*n* = 20), second immunization (E) (*n* = 20), and the day of farrowing (F) (*n* = 44) in Farm B. Correlations of serum and colostrum IgG levels at the first immunization (G) (*n* = 50) and the day of farrowing (H) (*n* = 50) in Farm C. (I) Correlations of serum and colostrum IgG levels on the day of farrowing in Farm D (*n* = 34). “*r*” values represented correlation coefficients. “*p*” values meant statistical significance.

The correlation between serum IgG and colostrum IgG varied across the different immunization regimens. During the first immunization, moderately strong correlations were observed in Farms A (*r* = 0.5654, *p* < 0.0001) and C (*r* = 0.5959, *p* < 0.0001), whereas the correlation in Farm B was poor and not statistically significant (*r* = 0.2178, *p* > 0.05) (Figure 4A, G, D). Following the second immunization, the correlation in Farm A remained moderately strong (*r* = 0.6051, *p* < 0.0001) (Figure 4B), whereas Farm B exhibited a poor and nonsignificant correlation (*r* = 0.2140, *p* > 0.05) (Figure 4E). On the day of farrowing, the correlation further increased in Farms A (*r* = 0.6853, *p* < 0.0001) and C (*r* = 0.8682, *p* < 0.0001), and was also moderately strong in Farm D (*r* = 0.7737, *p* < 0.0001). In contrast, Farm B continued to show a poor and nonsignificant correlation (*r* = 0.1614, *p* > 0.05) (Figure 4C, F, H, I).

### 3.5. Correlation Between Oral Swab and Colostrum IgA Antibodies

**Figure 5:**
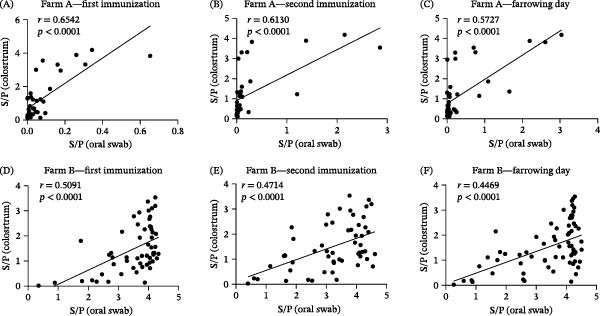
Correlations between oral swab and colostrum IgA antibodies. Correlations between oral swab and colostrum IgA at the first immunization (A) (*n* = 40), second immunization (B) (*n* = 40) and the day of farrowing (C) (*n* = 40) in Farm A. Correlations between oral swab and colostrum IgA at the first immunization (D) (*n* = 20), second immunization (E) (*n* = 20), and the day of farrowing (F) (*n* = 44) in Farm B. ‘‘*r*” values represented correlation coefficients. “*p*” values meant statistical significance.

In Farm A, oral swab IgA levels exhibited moderately strong correlations with colostrum IgA during the first (*r* = 0.6542) and second immunizations (*r* = 0.6130), whereas a fair correlation was observed at farrowing (*r* = 0.5727) (Figure 5A–C). All correlations were statistically significant (*p* < 0.0001). However, correlations in Farm B exhibited only fair correlation, with coefficients of 0.5091, 0.4714, and 0.4469 across the corresponding stages (Figure 5D–F). All correlations were statistically significant (*p* < 0.0001).

### 3.6. Correlation Between Rectal Swab and Colostrum IgA Antibodies

**Figure 6:**
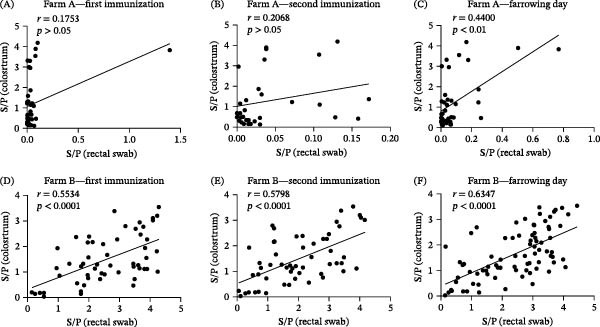
Correlations between rectal swab and colostrum IgA antibodies across different immunization stages. Correlations between rectal swab and colostrum IgA at the first immunization (A) (*n* = 40), second immunization (B) (*n* = 40), and the day of farrowing (C) (*n* = 40) in Farm A. Correlations between rectal swab and colostrum IgA at the first immunization (D) (*n* = 20), second immunization (E) (*n* = 20), and the day of farrowing (F) (*n* = 44) in Farm B. “*r*” values represented correlation coefficients. “*p*” values meant statistical significance.

In Farm A, rectal swab IgA levels showed poor and nonsignificant correlations with colostrum IgA during the first (*r* = 0.1753, *p* > 0.05) and second immunizations (*r* = 0.2068, *p* > 0.05). However, on the day of farrowing, rectal swab IgA exhibited a fair correlation with colostrum IgA (*r* = 0.4400, *p* < 0.01) (Figure 6A–C). In Farm B, the correlation between rectal swab and colostrum IgA increased following successive immunizations, peaking on the day of farrowing (*r* = 0.6347, *p* < 0.0001) (Figure 6D–F).

## 4. Discussion

Because the immune system of newborn piglets is not fully developed, PEDV infection can lead to death within a short period, with mortality rates reaching up to 100% [3], resulting in significant economic losses to the swine industry. Maternal antibodies are crucial for protecting piglets from infection [12, 13, 21]. In practice, sows are routinely immunized to enable piglets to acquire immunity through milk. Measurement of PEDV‐specific IgA antibodies in milk is the most direct and reliable indicator of neonatal resistance. However, antibody assessment in milk does not provide an early indication of the sow’s immune status, and by the time insufficient antibody levels are detected, the optimal intervention window is often missed. Therefore, this study systematically analyzed the correlations among PEDV antibodies in serum, oral swabs, rectal swabs, and colostrum collected from sows at different immunization stages to identify an early, reliable predictor of colostral antibody levels and provide guidance for effective PEDV prevention and control.

The utility of prepartum samples for predicting colostral antibody levels depends critically on the induction and sustained presence of antibodies. Our study directly addressed this by first characterizing the long‐term antibody kinetics in piglets. We confirmed a sustained antibody response, with both IgA and IgG remaining detectable for up to 31 weeks postinfection. Although conducted in piglets, this provides a critical reference point, as the more mature immune system of sows would be expected to support an even more prolonged antibody persistence. This confirmed durability not only justifies the temporal window for prepartum sampling but, more importantly, forms the essential logical foundation for our subsequent correlation analysis in sows.

By measuring PEDV antibodies in serum, oral swabs, and rectal swabs collected at multiple prepartum time points and in postpartum colostrum from sow herds with different immunization backgrounds and conducting correlation analyses, we identified a clear pattern. For PEDV‐specific IgG, only prepartum serum exhibited a significant correlation with colostrum, whereas oral and rectal swabs collected before farrowing, which had low antibody levels, exhibited poor correlation with colostrum (Figure S1). However, for PEDV‐specific IgA, serum, oral swabs, and rectal swabs collected at different immunization stages all exhibited varying degrees of correlation with postpartum colostrum. Among these sample types, prepartum serum and postpartum colostral PEDV IgA levels exhibited the strongest correlation, followed by oral swabs, whereas the rectal swabs exhibited the weakest correlation. The observed correlations between prepartum antibody levels in various maternal samples and postpartum colostral antibodies are best explained by the differential pathways governing IgG and IgA transfer to milk. For IgA, the strong correlations are consistent with the gut–mammary gland axis. This pathway dictates that antigen‐specific IgA^+^ B cells, activated in the intestinal mucosa, home to the mammary gland to produce sIgA in colostrum [10]. Thus, PEDV‐specific IgA in serum and in mucosal swabs both serve as predictive indicators for colostral sIgA output, with serum IgA reflecting circulating cells and mucosal swab IgA reflecting local induction. In contrast, the route for IgG is distinct. The correlation between serum IgG and colostral IgG is attributed to the passive transfer of systemic antibodies, a process independent of mucosal trafficking. Conversely, the very low levels and poor correlation of IgG in mucosal swabs with colostrum result from the fact that mucosal IgG likely represents minor local transudation and is not actively channeled through the gut–mammary axis.

These findings indicate that, particularly in sows with a history of PEDV infection or feedback, the immune status of sows can be effectively assessed by measuring PEDV‐specific IgA antibodies in prepartum serum, oral swabs, and rectal swabs. Compared with serum collection, oral and rectal swabs offer greater convenience, reduce animal stress, and improve biosafety by minimizing the risk of pathogen transmission through blood. Notably, higher dilution ratios for samples with high antibody levels resulted in stronger correlation and allowed more accurate discrimination of antibody levels across different immunization backgrounds and sample types. Thus, in practical application, dilution factors should be flexibly adjusted to ensure reliable evaluation.

In addition to demonstrating marked correlations between PEDV‐specific IgA in prepartum sow samples and postpartum colostrum across herds with diverse immunization backgrounds, we also observed substantial differences in PEDV IgA antibody levels among these herds. Notably, only herds exposed to feedback or natural infection developed high PEDV‐specific IgA levels, whereas vaccine‐immunized herds exhibited relatively low IgA levels and low antibody‐positive rates (Farm A). This disparity may explain why vaccine‐induced immunity is often reported as suboptimal in clinical settings, whereas feedback is perceived as more effective. Although feedback elicits robust immune responses in sows and enhances piglet protection, it also increases the risk of PEDV transmission and prolonged viral shedding within the herd. As maternal antibodies wane, piglets may become infected and develop diarrhea around the weaning period (Farms B and C). Naturally infected sows in late gestation can rapidly develop PEDV‐specific IgA and IgG antibodies; however, because they are simultaneously undergoing peak stages of viral infection and shedding, their piglets—despite receiving high maternal antibodies—are also exposed to high viral loads, leading to disease (Farm D). These observations indicate that feedback should be avoided in late‐gestation sows. For herd safety, feedback should primarily target replacement gilts, which should be introduced into the breeding herd population only after achieving high IgA levels and testing negative for PEDV. When emergency feedback of pregnant sows is unavoidable, the optimal timing should allow sufficient development of high levels of colostral IgA at farrowing and ensure minimal or absent viral shedding. Based on the antibody kinetics after infection, the optimal timing for feedback is approximately 4 weeks before farrowing.

In conclusion, this study further elucidated the IgG and IgA antibody response patterns following PEDV infection in pigs and demonstrated significant correlations between prepartum samples and postpartum colostrum, especially in sows with prior PEDV infection or feedback. These findings provide a robust basis for earlier herd PEDV immune assessment via prepartum IgA measurement, supporting more accurate sow vaccine efficacy evaluation, immunization optimization, and extended colostral antibody enhancement to improve maternal immunity and reduce piglet morbidity, mortality, and economic losses.

## Author Contributions

Ping Chen and Wenlong Zhao performed the lab work. Ping Chen, Wenlong Zhao, and Meng Ge analyzed the data, discussed the results, and wrote the manuscript. Ruide Deng, Jie Fan, Mei Huang, Yirun Tai, Mengyao Lu, Zhongxin Fan, Runcheng Li, and Wei Dong participated in the study design, animal husbandry, and sample processing.

## Funding

The study was supported by the Hunan Provincial Key Research and Development Program (Grant 2023NK2017), the National Natural Science Foundation of China (Grant 32072871), and the Hunan Provincial Special Program for Animal Disease Monitoring and Epidemiological Investigation.

## Disclosure

All authors read and approved the final manuscript.

## Ethics Statement

The animal study was reviewed and approved by the Animal Experiments Ethics Committee of Hunan Agricultural University. All procedures were done by strictly following the Experimental Animal Care and Use Guidelines of the Ministry of Science and Technology of China (MOST‐2011‐02).

## Conflicts of Interest

The authors declare no conflicts of interest.

## Supporting Information

Additional supporting information can be found online in the Supporting Information section.

## Supporting information


**Supporting Information** Figure S1 shows the correlations of IgG antibody levels in oral and rectal swabs with colostrum IgG levels.

## Data Availability

The data supporting the findings of this study are available from the corresponding author upon reasonable request.

## References

[1] Antas M. and Woźniakowski G. , Current Status of Porcine Epidemic Diarrhoea (PED) in European Pigs, Journal of Veterinary Research. (2019) 63, no. 4, 465–470, 10.2478/jvetres-2019-0064. 31934654 PMC6950429

[2] Wang X. , Niu B. , and Yan H. , et al.Genetic Properties of Endemic Chinese Porcine Epidemic Diarrhea Virus Strains Isolated since 2010, Archives of Virology. (2013) 158, no. 12, 2487–2494, 10.1007/s00705-013-1767-7, 2-s2.0-84887934820. 23797760 PMC7087078

[3] Alvarez J. , Sarradell J. , Morrison R. , and Perez A. , Impact of Porcine Epidemic Diarrhea on Performance of Growing Pigs, PLoS ONE. (2015) 10, 10.1371/journal.pone.0120532, 2-s2.0-84929492963. PMC4359118 25768287

[4] Chen J.-F. , Sun D.-B. , and Wang C.-B. , et al.Molecular Characterization and Phylogenetic Analysis of Membrane Protein Genes of Porcine Epidemic Diarrhea Virus Isolates in China, Virus Genes. (2008) 36, no. 2, 355–364, 10.1007/s11262-007-0196-7, 2-s2.0-41049117215. 18214664 PMC7088904

[5] Pyrc K. , Berkhout B. , and van der Hoek L. , The Novel Human Coronaviruses NL63 and HKU1, Journal of Virology. (2007) 81, no. 7, 3051–3057, 10.1128/JVI.01466-06, 2-s2.0-33947376439. 17079323 PMC1866027

[6] Chang S.-H. , Bae J.-L. , and Kang T.-J. , et al.Identification of the Epitope Region Capable of Inducing Neutralizing Antibodies Against the Porcine Epidemic Diarrhea Virus, Molecules and Cells. (2002) 14, no. 2, 295–299, 10.1016/S1016-8478(23)15106-5. 12442904

[7] Liu J. , Shi H. , and Chen J. , et al.Neutralization of Genotype 2 Porcine Epidemic Diarrhea Virus Strains by a Novel Monoclonal Antibody, Virology. (2017) 507, 257–262, 10.1016/j.virol.2017.04.026, 2-s2.0-85018340479. 28463713 PMC7172788

[8] Stevenson G. W. , Hoang H. , and Schwartz K. J. , et al.Emergence of *Porcine epidemic diarrhea virus* in the United States: Clinical Signs, Lesions, and Viral Genomic Sequences, Journal of Veterinary Diagnostic Investigation. (2013) 25, no. 5, 649–654, 10.1177/1040638713501675, 2-s2.0-84884153028. 23963154

[9] Zhang J. , Zhao D. , and Yi D. , et al.Microarray Analysis Reveals the Inhibition of Intestinal Expression of Nutrient Transporters in Piglets Infected With Porcine Epidemic Diarrhea Virus, Scientific Reports. (2019) 9, no. 1, 10.1038/s41598-019-56391-1, 19798. 31875021 PMC6930262

[10] Chattha K. S. , Roth J. A. , and Saif L. J. , Strategies for Design and Application of Enteric Viral Vaccines, Annual Review of Animal Biosciences. (2015) 3, no. 1, 375–395, 10.1146/annurev-animal-022114-111038, 2-s2.0-84923207326. 25387111

[11] Salmon H. , Berri M. , Gerdts V. , and Meurens F. , Humoral and Cellular Factors of Maternal Immunity in Swine, Developmental & Comparative Immunology. (2009) 33, no. 3, 384–393, 10.1016/j.dci.2008.07.007, 2-s2.0-57149121074. 18761034

[12] Langel S. N. , Paim F. C. , and Alhamo M. A. , et al.Stage of Gestation at Porcine Epidemic Diarrhea Virus Infection of Pregnant Swine Impacts Maternal Immunity and Lactogenic Immune Protection of Neonatal Suckling Piglets, Frontiers in Immunology. (2019) 10, 10.3389/fimmu.2019.00727, 2-s2.0-85065779756, 727. 31068924 PMC6491507

[13] Poonsuk K. , Giménez-Lirola L. G. , and Zhang J. , et al.Does Circulating Antibody Play a Role in the Protection of Piglets Against Porcine Epidemic Diarrhea Virus?, PLoS ONE. (2016) 11, 10.1371/journal.pone.0153041, 2-s2.0-84962852734. PMC4822964 27050556

[14] Saif L. J. , Enteric Viral Infections of Pigs and Strategies for Induction of Mucosal Immunity, Advances in Veterinary Medicine. (1999) 41, 429–446, 10.1016/S0065-3519(99)80033-0, 2-s2.0-0032621003. 9890034 PMC7149314

[15] Niederwerder M. C. and Hesse R. A. , Swine Enteric Coronavirus Disease: A Review of 4 Years With Porcine Epidemic Diarrhoea Virus and Porcine Deltacoronavirus in the United States and Canada, Transboundary and Emerging Diseases. (2018) 65, no. 3, 660–675, 10.1111/tbed.12823, 2-s2.0-85041177518. 29392870 PMC7169865

[16] Boonsoongnern P. , Boodde O. , and Chumsing W. , et al.Correlation Between Antibody Response Against Porcine Epidemic Diarrhea Virus in Sows and Their Offspring Under Field Conditions, Veterinary World. (2021) 14, 1689–1694, 10.14202/vetworld.2021.1689-1694. 34316220 PMC8304444

[17] Hu Z. , Li Y. , and Zhang B. , et al.Serum IgA Antibody Level Against Porcine Epidemic Diarrhea Virus Is a Potential Pre-Evaluation Indicator of Immunization Effects in Sows During Parturition under Field Conditions, Porcine Health Management. (2024) 10, no. 1, 10.1186/s40813-024-00382-w, 32. 39228006 PMC11373460

[18] Ouyang K. , Shyu D.-L. , and Dhakal S. , et al.Evaluation of Humoral Immune Status in Porcine Epidemic Diarrhea Virus (PEDV) Infected Sows Under Field Conditions, Veterinary Research. (2015) 46, no. 1, 10.1186/s13567-015-0285-x, 2-s2.0-84951064820, 140. 26667229 PMC4699368

[19] Rui-de D. , Zhi-xiong C. , and Ze-qi H. , et al.Study on the Correlation between IgA in Serum and Colostrum of Sows Based on PEDV IgA Indirect ELISA Method, Chinese Journal of Preventive Veterinary Medicine. (2024) 46, 1245–1254, 10.3969/j.issn.1008-0589.202403003.

[20] Chan Y. H. , Biostatistics丨04: Correlational Analysis, Singapore Medical Journal. (2003) 44, 614–619. 14770254

[21] Leidenberger S. , Schröder C. , and Zani L. , et al.Virulence of Current German PEDV Strains in Suckling Pigs and Investigation of Protective Effects of Maternally Derived Antibodies, Scientific Reports. (2017) 7, no. 1, 10.1038/s41598-017-11160-w, 2-s2.0-85029142517, 10825. 28883628 PMC5589859

