# Supplementary material for: Dynamic Antibody Responses in Porcine Epidemic Diarrhea Virus‐Infected Pigs and Correlation of Prepartum Serum, Oral Swabs, and Rectal Swabs With Postpartum Colostral IgA and IgG in Sows
Source: Transbound Emerg Dis. 2026 May 26;2026:6411953. doi: 10.1155/tbed/6411953 (PMC13202205; doi:10.1155/tbed/6411953)
Supplement: Supplementary file 1 — Supporting Information Figure S1 shows the correlations of IgG antibody levels in oral and rectal swabs with colostrum IgG levels. [file TBED-2026-6411953-s001.docx]

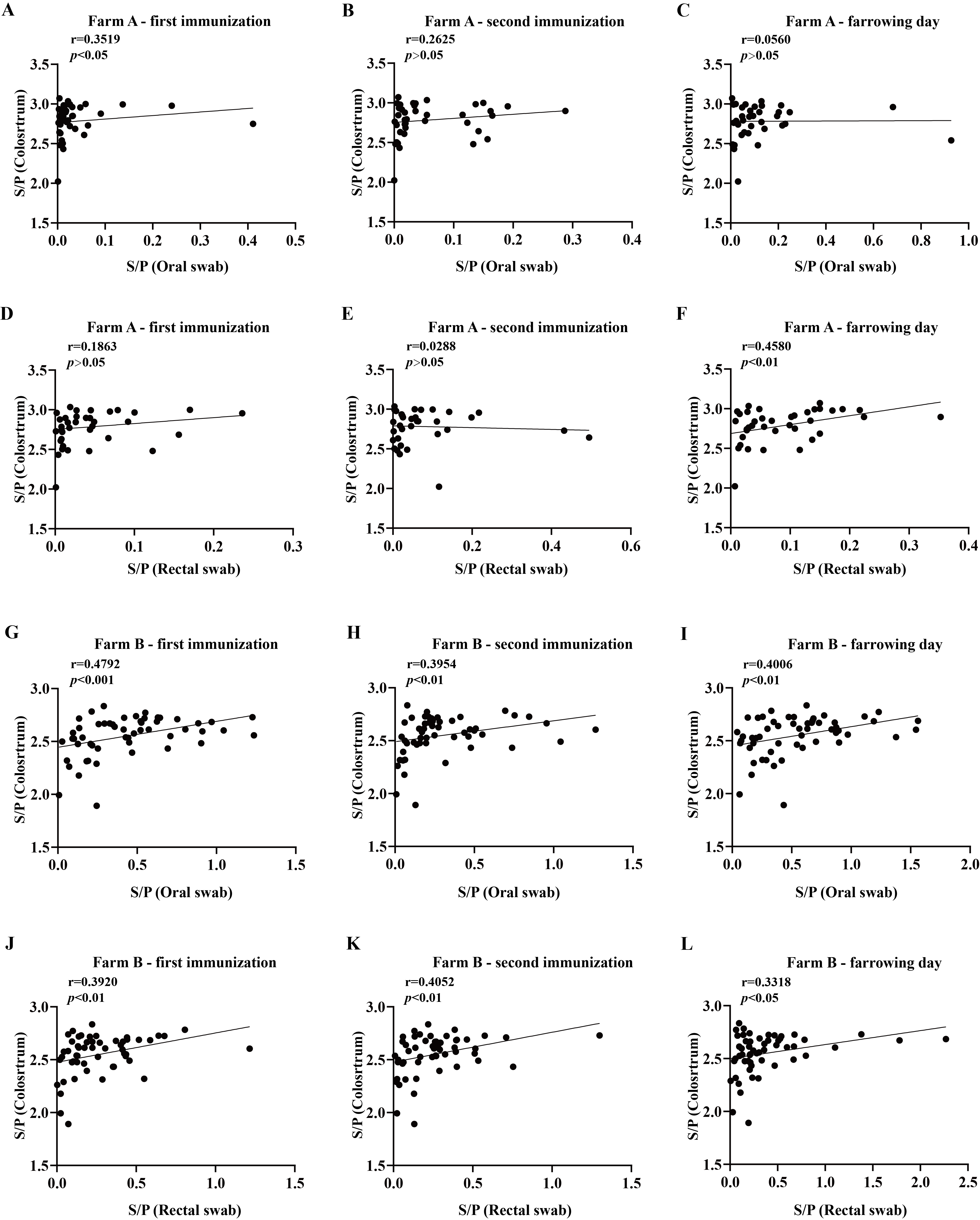


Supplementary Figure 1. Correlations of IgG antibody levels in oral and rectal swabs with colostrum IgG levels. Correlations between oral swab and colostrum IgG at the first immunization (A), second immunization (B) and the day of farrowing (C) in Farm A. Correlations between rectal swab and colostrum IgG at the first immunization (D), second immunization (E), and the day of farrowing (F) in Farm A. Correlations between oral swab and colostrum IgG at the first immunization (G), second immunization (H), and the day of farrowing (I) in Farm B. Correlations between rectal swab and colostrum IgA at the first immunization (J), second immunization (K), and the day of farrowing (L) in Farm B. ‘‘r’’ values represented correlation coefficients. “*p*” values meant statistical significance.
